# Supplementary material for: Bamboo Plant Part Preference Affects the Nutrients Digestibility and Intestinal Microbiota of Geriatric Giant Pandas
Source: Animals (Basel). 2023 Feb 25;13(5):844. doi: 10.3390/ani13050844 (PMC10000146; doi:10.3390/ani13050844)
Supplement: Supplementary file 1 [file animals-13-00844-s001.zip › animals-2171654-supplementary.pdf]

animals

MDPI

Table S1. The dominant phyla (average abundance >1%) in different groups.

Treatment	Phylum	Average Abundance
AS	*Firmicutes*	42.85%
	Proteobacteria	52.92%
AL	*Firmicutes*	62.84%
	Proteobacteria	33.01%
OS	*Firmicutes*	50.69%
	Proteobacteria	46.91%
OL	*Firmicutes*	51.04%
	Proteobacteria	44.18%

AS, adult giant panda fed with bamboo shoots; AL, adult giant panda fed with bamboo leaves; OS, old giant panda fed with bamboo shoots; OL, old giant panda fed with bamboo leaves.

Table S2. The dominant genera (average abundance >1%) in different groups.

Treatment	Phylum	Average Abundance
AS	*Escherichia-Shigella*	33.78%
	Clostridium_sensu_stricto_1	23.29%
	Streptococcus	1.38%
	Lactococcus	8.98%
	Turicibacter	2.39%
	Raoultella	4.43%
	Citrobacter	3.66%
	Enterococcus	2.34%
	Ralstonia	2.71%
	Pantoea	2.42%
	Cellulosilyticum	2.30%
AL	*Escherichia-Shigella*	30.66%
	Clostridium_sensu_stricto_1	46.23%
	Streptococcus	10.27%
	Terrisporobacter	1.87%
OS	*Escherichia-Shigella*	28.17%
	Clostridium_sensu_stricto_1	24.62%
	Streptococcus	2.06%
	Lactococcus	9.67%
	Turicibacter	5.22%
	Raoultella	3.10%
	Citrobacter	3.29%
	Enterococcus	3.40%
	Ralstonia	2.09%
	Pantoea	2.01%
	Cellulosilyticum	1.95%
OL	*Escherichia-Shigella*	45.82%
	Clostridium_sensu_stricto_1	34.06%
	Streptococcus	9.89%
	Turicibacter	1.10%
	Terrisporobacter	1.53%

AS, adult giant panda fed with bamboo shoots; AL, adult giant panda fed with bamboo leaves; OS, old giant panda fed with bamboo shoots; OL, old giant panda fed with bamboo leaves.
